# Digital Mental Health Screening, Feedback, and Referral System for Teens With Socially Complex Needs: Protocol for a Randomized Controlled Trial Integrating the Teen Assess, Check, and Heal System into Pediatric Primary Care

**DOI:** 10.2196/65245

**Published:** 2025-02-18

**Authors:** Colleen Stiles-Shields, Gabriella Bobadilla, Karen Reyes, Erika L Gustafson, Matthew Lowther, Dale L Smith, Charles Frisbie, Camilla Antognini, Grace Dyer, Rae MacCarthy, Nicolò Martinengo, Guy Morris, Alissa Touranachun, Kimberlee M Wilkens, Wrenetha A Julion, Niranjan S Karnik

**Affiliations:** 1 Institute for Juvenile Research Department of Psychiatry University of Illinois Chicago Chicago, IL United States; 2 AI.Health4All Center College of Medicine University of Illinois Chicago Chicago, IL United States; 3 Department of Psychiatry Rush University Medical Center Chicago, IL United States; 4 Innovation Center University of Illinois Chicago Chicago, IL United States; 5 Children and Family Nursing Department of Women RUSH University Medical Center Chicago, IL United States

**Keywords:** teens, primary care, digital mental health, low intensity treatments, disparities

## Abstract

**Background:**

Teens with socially complex needs—those who face multiple and potentially overlapping adversities—are disproportionately affected by several barriers to mental health screening and treatment. Pediatric primary care (PPC) is a typically low-stigmatized setting for teens that is visited at least annually. As such, implementing digital mental health tools (DMH), as low-intensity treatments in PPCs may increase the reach of such tools for teens with socially complex needs.

**Objective:**

This study aimed to evaluate the Teen Assess, Check, and Heal (TeACH) System in comparison to a control condition while integrated into PPCs at 2 Medical Centers serving teen patients in Chicago, Illinois. Through collaboration with key players throughout the design and implementation planning phases, the TeACH System is hypothesized to increase teen patient self-reported engagement with DMH and address specific individual-level barriers to mental health care, compared with a digital psychoeducation control condition.

**Methods:**

Eligible participants will be recruited through PPC clinics housed within the University of Illinois Chicago (UIC) and Rush University Medical Center (RUSH). Recruitment involves invitations from research staff members and primary care clinicians and staff members, as well as posting flyers with QR codes at the specified clinics. All participants complete a brief demographic survey, baseline survey, and Kiddie-Computerized Adaptive Tests Anxiety Module. Participants are randomized to receive either the control condition (digital evidence-based workbook) or the intervention (TeACH System Feedback and Resources). All randomized participants will then be invited to complete an immediate and 1-week follow-up survey. The primary outcomes assess changes in engagement with DMH (ie, likelihood to use DMH for anxiety and actual DMH use) and individual-level barriers to mental health care (ie, symptom understanding and confidence to act). Descriptive analyses will be conducted to characterize the sample and usability ratings of the TeACH System. Linear or generalized linear mixed effects regression models will examine differences in primary outcomes over time.

**Results:**

Recruitment began in July 2024 and data collection is expected to be completed by August 2025. To date, 122 teens have assented to complete study activities, 80 have been randomized (an additional 24 teens have had subthreshold anxiety symptoms and were therefore not randomized), and 42 teens have completed the 1-week follow-up assessment.

**Conclusions:**

This study will provide preliminary feasibility data that may inform how the TeACH System and other DMH low-intensity treatments might better engage and support teens with socially complex needs.

**Trial Registration:**

ClinicalTrials.gov NCT05466929; https://clinicaltrials.gov/study/NCT05466929

**International Registered Report Identifier (IRRID):**

DERR1-10.2196/65245

## Introduction

### Background

Even before the multiple endemics occurring since 2020, pediatric mental health disorders and symptoms were the leading causes of disability and negative sequelae for youth [1]. For some time, mental health disorders have been the most common disease of childhood in the United States, surpassing the *combined* rates of multiple pediatric conditions (eg, cancer and diabetes) [2-4]. Despite the tremendous impact of mental health disorders on teens, millions do not receive mental health care [2]. Anxiety is particularly untreated, as up to 80% of youth with a diagnosable anxiety disorder do not receive mental health care [2,3,5]. Most exposed to this failure of mental health care are teens with socially complex needs, those who face multiple and potentially overlapping adversities, such as (1) enduring adverse childhood experiences, (2) residing in a systemically excluded community that experiences disproportionate disparities in health outcomes and health care access, or (3) being minoritized due to socioeconomic status as well as racial, ethnic, gender, or sexual identity or identities [1,6-8]. Teens with socially complex needs have multiple barriers placed between them and mental health care, which the current system is failing to address [9-15]. As such, traditional methods for reaching such teens are not working, resulting in life-long health disparities and a significant public health impact [2,6,7,16,17].

### Digital Tools Implemented in Primary Care to Extend Care Capacity

Pediatric primary care (PPC) is one setting with strong potential as an environment to engage and screen teens with socially complex needs. Indeed, nearly all youth visit a PPC office annually for well-child visits as well as illnesses or injuries that necessitate care (eg, ear infection) [18]. As such, PPC is a centralized and typically low-stigmatized setting for teens of all backgrounds. Mental health care is increasingly warranted in PPC, with the American Academy of Pediatrics recommending universal anxiety screening for all children and adolescents, aged 8-18 years, during PPC visits [19]. However, PPC providers report variable administration of standardized assessment measures [20] and multiple barriers to mental health screening, broadly [20-22]. Further, when mental health needs, such as anxiety, are screened or arise in PPC, pediatricians report multiple barriers to assessing mental health needs and connecting patients to therapy [23,24], adding to the obstacles to care noted above. Even in the case of using a collaborative care model for depression in primary care [25], a pragmatic approach for anxiety that embraces the logistical realities that providers face in delivering mental health screening and ensuing recommendations is lacking [26,27]. A potential solution to these barriers is to incorporate digital mental health tools (DMH) as a low-intensity treatment (LIT) into PPCs. LITs are patient-facing tools that may provide screening, psychoeducation, resources, or combinations of these items. Given the long-standing efficacy of LITs in digital and other delivery mechanisms for mild to moderate symptom presentations (eg, self-help books) [28], DMH LITs have been proposed as a scalable and strategic first-line model of care in PPC and similar care environments [29].

Integrating DMH LITs into spaces that teens already visit, such as PPC, has the potential to increase mental health care reach. However, uptake and subsequent clinical outcomes will not be impacted if teens do not engage with DMH in their daily lives [30]. One problem is a lack of implementation planning early on in the design processes for DMH [31,32]. Also implicated in poor engagement is a failure to address the range of multilevel implementation barriers, spanning from individual-level barriers (eg, problem recognition, stigma, and confidence to act), intervention barriers (eg, usability and relevance), and systemic barriers (eg, accessibility and cost) [24-27,33]. Interventions such as incorporating motivational interviewing [34] and usage incentivizes [35-37] have improved DMH engagement, but diminish scalability [38]. As such, means to target barriers must be identified and harnessed to address poor engagement, the perennial failure of DMH deployment to date.

### Objectives

In this study, human-centered design methodologies and an implementation science framework [39-41] were used to guide the development and implementation of the Teen Assess, Check, and Heal (TeACH System). Through the use of these methodologies and frameworks, the TeACH System was designed to target individual-level barriers to care (eg, feedback to directly respond to problem recognition, stigma, and confidence to act on symptoms), DMH intervention barriers (eg, human-centered design methodologies to optimize usability and relevance), and systemic barriers to care (eg, increase accessibility by integrating in a space already visited by teens, remove cost barrier by providing a freely accessible service and resources) [24-27,33]. Our primary aim was to increase DMH engagement for teens with socially complex needs through: (1) engaging teens and caregivers from the systemically-excluded and underserved communities of Chicago, Illinois, to refine the TeACH System to be engaging, appropriate, and in line with cultural and user needs; and (2) integrating the TeACH System into PPCs serving teens on the West Side of Chicago. The West Side of Chicago was selected for initial implementation planning and integration evaluations as part of a larger body of research focusing on adapting DMH LITs for and with teens living in communities disproportionately inflicted with health disparities, violence exposure, and higher economic hardship (eg, [42]). Through collaboration with key players throughout the design and implementation planning phases, the TeACH System was hypothesized to increase teen patient self-reported engagement with DMH and address specific individual-level barriers to mental health care, compared with a digital psychoeducation control condition. This hypothesis is being evaluated through a pilot feasibility randomized controlled trial in Chicago PPCs, comparing engagement outcomes for teens with anxiety randomized to use the full TeACH System to those who are provided access to a publicly available, digital evidence-based workbook for teens (psychoeducation control) [43,44].

## Methods

### Positionality

Positionality influences all aspects of a research study and is connected to the researchers’ personal and philosophical views [45]. In recognition of this influence, the positionality of the lead author of this study has been reported elsewhere [23] and is similarly described: (1) author CSS is a lifelong Chicagoan, (2) trained social worker, and (3) a pediatric psychologist who believes that engaging youth with DMH and in health environments they already frequent maybe 1 path to get more resources directly to youth to use as they need and want, but that DMH will not become a panacea to disparities. As a monolingual, White woman trained in health care settings, she has biases and experiences that may impact her interpretation of findings (eg, she is comfortable in health settings; DMH has primarily been tested with individuals who have overlapping identities with her [eg, college-educated and White women] [46]). To minimize bias, author CSS partnered with key players, by establishing interdisciplinary mentor and peer collaborator teams, to learn with teens, caregivers, and providers about their wants and needs for DMH in health care settings.

### Procedures

#### Study Design

**Figure 1:**
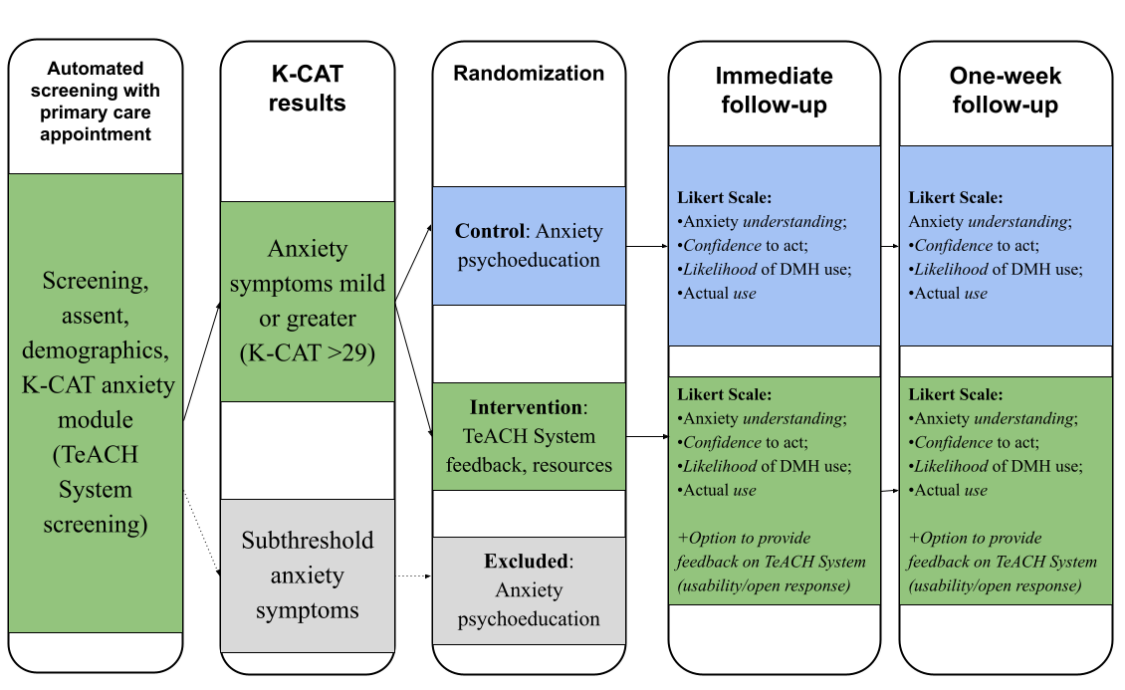
Participant flow through TeACH system trial in pediatric primary care K-CAT: Kiddie-Computerized Adaptive Tests.

**Figure 2:**
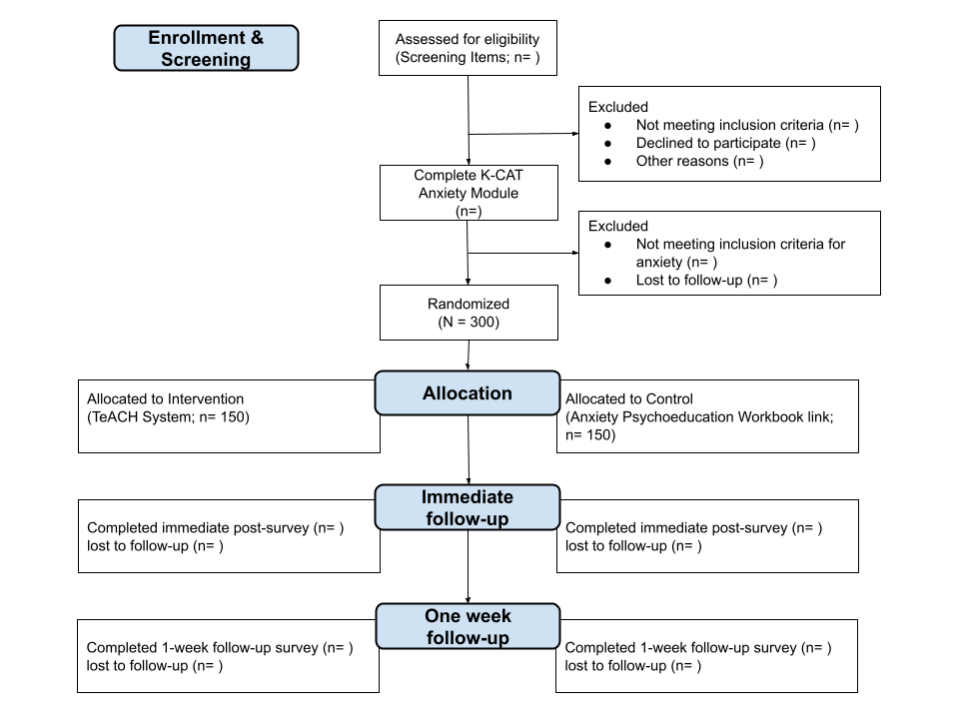
Study flow diagram.

The study design is a pilot feasibility randomized controlled trial. The participant focus is teen patients in PPCs with anxiety. While universal anxiety screening is recommended [19], pediatric anxiety has a long-standing history of being underrecognized and often untreated [47-49]. Participants first answer initial screening eligibility questions (ie, age, clinic location, and ability to complete and read surveys in English), followed by completing a digital assent. While a waiver of guardian consent was obtained from the institutional review board, participants are able to “opt in” to have a guardian provide consent in English or Spanish, should they elect to do so. Following assent and optional guardian permission, participants complete a brief demographic survey, indicate their preference for future contact (ie, text message or email), and complete a brief assessment of the primary outcomes (Measures section). All participants then complete the Kiddie-Computerized Adaptive Tests (K-CAT) Anxiety Module [50] to ensure eligibility (ie, a score >29 and indicating mild or greater anxiety symptoms). Participants who do not meet these criteria are excluded and offered access to a publicly available, digital evidence-based workbook for teens [43,44]. Participants who do meet the criteria for mild or greater symptoms of anxiety are randomized into either the control arm (digital evidence-based workbook [43,44]) or the Intervention Arm (TeACH System Feedback and Resources). Randomization is automated through the Research Electronic Data Capture (REDCap, Vanderbilt University) platform, and study staff members are blind to allocation. Following a review of these resources, all participants are invited to complete an immediate follow-up survey. One week following this interaction, participants are invited to complete the same follow-up survey. Figures 1 and 2 display the study design flow.

#### Setting and Recruitment

Eligible participants are being recruited through PPC clinics housed within the University of Illinois Chicago (UIC) and Rush University Medical Center (RUSH). Both hospital systems’ primary campuses are situated on the West Side of Chicago and serve diverse patient populations [51,52]. Eligible participants are being recruited in clinics through (1) QR codes on displayed and circulated flyers, including sharing of the flyer by PPC pediatricians, trainees, and staff; and (2) invitation from research staff (ie, offered tablet in the clinic waiting room or while waiting in an exam room) or PPC clinicians and staff members.

#### Inclusion and Exclusion Criteria

For study inclusion, participants must be (1) receiving care at the specified clinics at UIC or RUSH; (2) between 13 and 17 years of age; and (3) able to speak and read in English. These inclusion criteria are determined by teen self-report (ie, answering 3 screening questions based on these criteria). Following assent and completion of a demographics and engagement survey (Measures section), final inclusion criteria are assessed based on responses to the K-CAT Anxiety Module. Namely, to be randomized, participants must also meet criteria for mild or greater symptoms of anxiety (ie, K-CAT Anxiety Module score >29) [50]. There are no assessments for comorbidities, nor any exclusion criteria beyond not meeting the inclusion criteria noted above.

#### TeACH System

**Figure 3:**
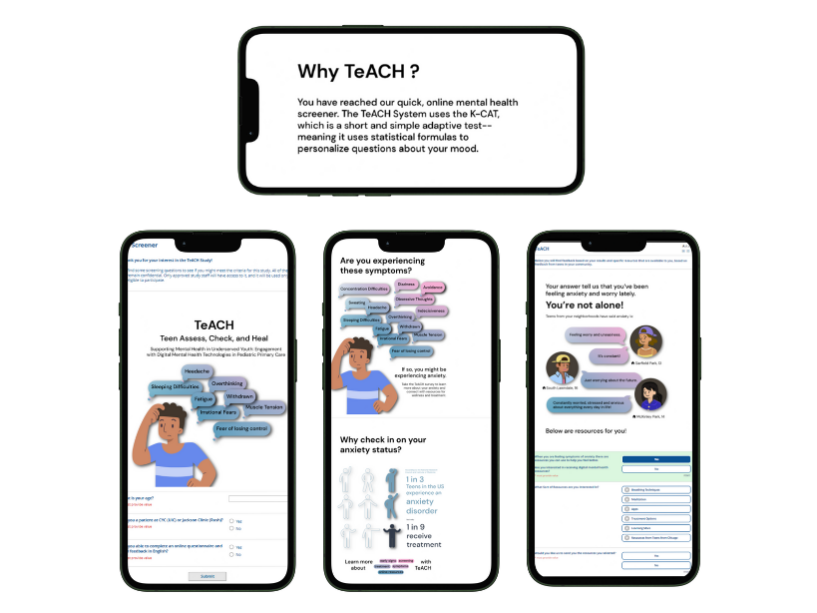
Sample screenshots of the TeACH system.

The TeACH System is a DMH LIT that includes teen patient-facing brief assessment, feedback, and resources for symptoms of anxiety (sample screenshots displayed in Figure 3. Increasing DMH engagement for teens with socially complex needs is the primary aim of the current iteration of the TeACH System. The mechanism to achieve this outcome includes the use of key player involvement throughout the design and implementation planning processes. Consistent with a broad spectrum of community-engaged research practices [53], involvement of teens and caregivers from the West and South Side Communities of Chicago have ranged from 1-time interviews and focus groups to co-design activities, with plans to form a community advisory board for the broader line of research led by this team [42,54,55]. Most relevant to the design of the current iteration of the TeACH System (eg, direct quotes described below), formative usability testing sessions were completed remotely with 10 teens (mean age 15.9, SD 0.99 years; female: n=8, 80.0%; Hispanic or Latino: n=6, 60.0%; Middle Eastern of North African: n=1, 14.3%; White: n=6, 60.0%) and 7 parents of teens (mean age 41.86, SD 8.7 years; female: n=5, 71.4%; Hispanic or Latiné: n=1, 14.3%; Middle Eastern or North African: n=1, 14.3%; Black or African American: n=4, 57.1%) from the West and South Side Communities of Chicago.

#### TeACH System Assessment

The TeACH System uses the Kiddie-Computerized Adaptive Tests (K-CAT) Anxiety Module to assess teen patient anxiety symptoms [50]. The K-CAT Anxiety Module (youth report; ages 7 to 17 years) uses an item bank of more than 200 items, typically offering 10 questions to a teen user to predict the likelihood of a diagnosis of generalized anxiety disorder (area under the receiver operating characteristic curve=0.83). However, the module is not limited to generalized anxiety disorder and may also be used for general anxiety screening in youth [50]. Future iterations of the TeACH System may use the full K-CAT (ie, assessing anxiety, depression, substance misuse, oppositional defiant disorder, attention-deficit/hyperactivity disorder, mania, conduct disorder, and suicidality), but the current version of the TeACH System is solely assessing anxiety. This decision was driven by input from PPC clinicians and staff members who expressed concern about follow-up mental health care, safety, and workflow disruptions around more expansive digital mental health assessments [23]. Participants in the current study are eligible for randomization with a score greater than 29 on the K-CAT Anxiety Module, indicating mild, or higher anxiety symptoms [50].

#### TeACH System Feedback

Following the completion of the K-CAT, the TeACH System informs users that they will be provided feedback and specific resources that were informed by feedback from “teens in your community.” The feedback provides an infographic that starts by stating: “Your answer tells us that you’ve been feeling anxiety and worry lately,” followed by the validation that “You’re not alone!” Following this, 4 quotes from Chicago teens describe anxiety as: “feeling worry and uneasiness”; “It’s constant!”; “Just worrying about the future”; and “Constantly worried, stressed, and anxious about every day in life!” before proceeding to viewing resources, users are prompted that “When you are feeling symptoms of anxiety, there are resources you can use to help you feel better.” In sum, the feedback section aims to briefly address multiple individual barriers to care, including problem recognition (ie, “Your answers tell us that you’ve been feeling anxiety and worry lately.”), stigma (ie, “You’re not alone!” and quotes from “Teens from your neighborhoods”), and confidence to act (ie, “When you are feeling symptoms of anxiety, there are resources you can use to help you feel better”) [24-26].

#### TeACH System Resources

Following the feedback, users are asked if they would like to receive DMH resources. The resources are all freely accessible online and are presented to participants through both sample screenshots and links to their direct sources (eg, App Store, YouTube, and TikTok). All resources are grounded in evidence-based skills (eg, progressive muscle relaxation and diaphragmatic breathing) and psychoeducation (including brief videos from teens and young adults with lived experience with anxiety). If participants indicate that they do not wish to receive DMH resources, they are asked to provide a reason as to why. If they would like to receive DMH resources, they are asked what types of resources they would prefer (eg, “Breathing Techniques,” “Apps,” and “Learning More”). This checkbox selection allows teens to view the content they wish, as opposed to getting all options at once, which can be overwhelming. Users may also opt to have the resources sent to them at their preferred method of contact (ie, email or text message).

#### TeACH System Platform

The TeACH System is intended to be adaptable and scalable. As such, using a simple but secure platform that may be easily edited at minimal cost was ideal. UIC’s instance of REDCap was therefore selected as the platform to build and manage the TeACH System [56]. REDCap is housed and managed by the University, as is common across multiple university and health care systems. To enhance the aesthetic appeal and engagement of the TeACH System for teen users, the UIC Innovation Center collaborated on its development within REDCap. An interdisciplinary team of researchers applied human-centered design techniques and analysis frameworks to effectively present the survey and maintain participant interaction throughout. They used user journey maps and extensive user testing to ensure a smooth and intuitive survey flow. The team also crafted a distinct visual language for the TeACH System, incorporating contemporary graphic elements inspired by popular social media platforms, calming color choices, and clear, simple language to appeal to the teen audience and put users at ease.

#### Control Condition

Following screening, assent, demographic characteristic assessment, and completion of the K-CAT Anxiety Module, participants randomized to the control condition receive different feedback and resources than those allocated to interact with the full TeACH System. The control condition includes immediate feedback about anxiety symptoms (ie, “Your answers tell us that you’ve been feeling anxiety and worry lately.”). Following this, participants are queried whether they would like access to a digital resource. If they respond negatively, they are prompted to provide their reasoning. If they respond positively, they are provided with a direct link to a free download of an evidence-based workbook for teens with anxiety [43,44].

### Measures

#### Demographics

Following assent, teens are asked to report the following demographic information: name, age, ethnicity (Hispanic or Latiné and Middle Eastern or North African), race, pronouns, gender, preferred language, the reason for visiting primary care, and preferred method for contact (ie, email or text message).

#### Primary Outcomes

The primary outcomes are administered within REDCap and are assessed at 3 time points for all participants: (1) baseline: following assent and before administration of the K-CAT; (2) post interaction: immediately after receiving the allocated feedback and resources; and (3) maintenance: 1 week following the interaction. The primary outcomes are discrete variables focusing on individual-level barriers to mental health care (ie, symptom understanding, confidence to act [24-26]) and engagement with DMH (ie, likelihood to use DMH for anxiety, actual DMH use). Namely, through a questionnaire created for this study, participants rate on a Likert scale (1=not at all to 5=completely): (1) anxiety *understanding* (ie, “I understand what anxiety is.”); (2) *confidence to act* (ie, “I feel confident I can do something if I feel anxiety.”); and (3) *likelihood* to use digital tools for anxiety (ie, “If I feel anxiety, I would use a digital tool to help (app, website, and reel or video).”). Following this, participants indicate whether they have used a digital tool for their anxiety before (ie, yes, no, and unsure).

#### Usability

Usability of the TeACH System and its control arm are assessed post interaction (ie, immediately after receiving the allocated feedback and resources) and 1 week after the interaction through the After Scenario Questionnaire, a 3-item measure of usability [57].

### Data Analyses

#### Overview

Descriptive analyses will be conducted to characterize the sample, primary outcomes, and usability ratings of the TeACH System. To assess changes in the engagement outcomes over time and account for missing data, linear or generalized linear mixed effects regression models will examine differences in responses to the baseline, postinteraction, and maintenance questionnaires. Demographic variables and existing DMH use, as well as their interactions with time, will be examined as covariates to assess differential engagement outcomes based on these variables.

#### Sample Size and Power

Power and sample size computations were based on previously published work [58,59]. Conservative estimates of sample sizes were estimated (ie, anticipating a 15% attrition rate on the follow-up survey), which indicated that 300 patients were deemed appropriate to examine the initial TeACH System use (ie, K-CAT Anxiety module, feedback, and resources) after being invited during their PPC appointment. With an expected 15% attrition rate for the follow-up questionnaire 1 week later, we would be adequately powered for within-subjects (pre vs post using the main effect of time) and between groups comparisons (TeACH System vs Control condition using treatment by time interaction). For within-subjects comparison, our proposed sample size is overpowered such that we should exceed 90% power to detect differences generally considered to be moderate in size (*d*=0.5) with a sample size exceeding approximately 70 participants. To detect within-subjects differences from baseline to endpoint equivalent to *d*=0.34 for the engagement outcomes, we would require a sample size of approximately 136 for 95% power. As such, our proposed sample is more than adequate for the planned within-subjects comparisons. For between-group comparisons with the anticipated 15% attrition rate, we should have 95% power to detect differences generally considered moderate in size (*d*=0.5) with 240 participants. With our proposed sample size of 300 participants, we will have 80% power to detect smaller differences equivalent to *d*=0.34.

### Ethical Considerations

All procedures were approved by the institutional review boards of the University of Illinois Chicago (2024-0252) and Rush University Medical Center (20051313). Further, ongoing study monitoring is being overseen by an independent Data Safety and Monitoring Board. All participants complete a digital assent and are compensated for completing a follow-up assessment with a US $15 Amazon e-gift code (see the section Study Design below).

## Results

This study received funding from the National Institute of Mental Health on September 13, 2021 (K08 MH125069), and began recruiting at UIC in July 2024 and at Rush in August 2024. Earlier funded activities informed the design and implementation planning for the current trial [23,32,42,54]. To date, 122 teens have assented to complete study activities, 80 have been randomized (an additional 24 teens have had subthreshold anxiety symptoms and were therefore not randomized), and 42 teens have completed the one-week follow-up assessment. We expect data collection to be completed by August 2025.

## Discussion

### Overview

The current feasibility study aims to evaluate the TeACH System in terms of (1) increasing engagement with DMH and (2) addressing individual-level barriers to mental health care. These outcomes are being examined in the context of integration in urban PPC clinics and in comparison to a control condition (ie, access to a digital workbook). The design and implementation plan for the TeACH System involved collaborative input from key players (ie, teens, caregivers, and PPC staff and clinicians) to increase the likelihood of teen engagement and acceptability in a dynamic health care setting [23,42,54].

The TeACH System stands as one of a growing number of DMH LITs being evaluated and implemented in care settings (eg, [60,61]). DMH LITs have demonstrated promise in increasing engagement for minoritized teens, compared with more intensive digital and traditional care models [37]. Amid this landscape, some unique aspects of the TeACH System situate it to adapt and scale well based on user and setting needs. First, the TeACH System is housed on a secure, stable, and editable platform (ie, REDCap [56]) and may therefore be adapted across iterations and for differing populations and settings. For example, screening may be expanded from self-reported anxiety only (current version) to up to 8 diagnostic categories assessed through the K-CAT (ie, anxiety, attention-deficit/hyperactivity disorder, conduct disorder, depression, mania, oppositional defiant disorder, substance use disorder, and suicidality [50]). Second, infographics and other design features may be changed to reflect the language and design preferences of different target groups. Similarly, resources may be edited or changed based on availability (eg, open access removal of a video), population preferences, and platform safety and availability (eg, if a “TikTok ban” results in higher teen use of a different platform). Finally, the TeACH System–and any future iterations are grounded in human-centered design with input from key players to target DMH engagement. Such targeting is hypothesized to serve as a mechanism of DMH to ultimately impact clinical outcomes [62]. In sum, the TeACH System has been designed for teens with both anxiety and socially complex needs while visiting primary care. However, future iterations may be easily adapted at any level for integration in settings youth visit regularly and trust.

### Future Directions

The scope of this study is associated with specific limitations and caveats, all of which inform future research directions. First, the current iteration of the TeACH System focuses solely on anxiety. While originally aimed to include the full K-CAT assessment, the scope was limited to anxiety to meet the implementation needs of PPC clinicians and staff members [23]. Anxiety was also selected to establish proof of concept for this first iteration, as anxiety is highly prevalent and socially minimized by clinicians and the public, making it less likely to be effectively screened and treated. Once the TeACH System establishes initial feasibility, it can be broadened to address multiple disorders through a larger study. Second, the K-CAT demonstrates the strongest accuracy when the parent proxy report and child self-report are integrated [50]. While the current iteration of the TeACH System is solely teen-facing, there is the potential for the TeACH System to have both caregiver and teen-facing elements in the future. Alternatively, summative feedback from this study may indicate that teens or families prefer for this to be a teen-facing tool, but with the option to inform trusted adults in their life about the indication of anxiety from the K-CAT. Further, the K-CAT is available and validated in both English and Spanish. As such, future iterations may also include cultural and linguistic adaptations to serve teens and caregivers who speak Spanish. Finally, while PPC is typically visited annually by most teens, integration in this care setting does not ensure that all teens will be reached. PPC was selected to establish feasibility for reasons detailed above, but future iterations may be adapted and integrated in other community spaces in which teens and families spend their time (eg, schools, parks, libraries, and churches).

### Conclusions

LITs have a long-standing history of benefiting individuals with mild to moderate mental health symptoms [28], implicating DMH LITs as a promising first-line approach in care settings [29]. The TeACH System represents 1 possible DMH LIT that may provide brief assessment, feedback, and resources for teen patients without unduly burdening a busy health care environment nor requiring the approval of a guardian to use. This study will provide preliminary feasibility data that may inform how the TeACH System and other DMH LITs might better engage and support teens with socially complex needs.

## Appendix

Multimedia Appendix 1 Peer-review report from the Mental Health Services Study Section, National Institute of Mental Health Initial Review Group (NIH).

## Abbreviations

DMH: digital mental health tools
K-CAT: Kiddie Computerized Adaptive Test
LIT: low-intensity treatment
PPC: pediatric primary care
REDCap: Research Electronic Data Capture
RUSH: Rush University Medical Center
TeACH System: Teen Assess, Check, and Heal System
UIC: University of Illinois Chicago
