# Supplementary material for: Digital Mental Health Screening, Feedback, and Referral System for Teens With Socially Complex Needs: Protocol for a Randomized Controlled Trial Integrating the Teen Assess, Check, and Heal System into Pediatric Primary Care
Source: JMIR Res Protoc. 2025 Feb 18;14:e65245. doi: 10.2196/65245 (PMC11888113; doi:10.2196/65245)
Supplement: Multimedia Appendix 1 [file resprot_v14i1e65245_app1.pdf]

1K08MH125069-01A1 STILES-SHIELDS, COLLEEN

RESUME AND SUMMARY OF DISCUSSION: This is a resubmission of a K08 application focused on the reduction of mental health disparities in pediatric primary care settings. The career development plan focuses on pediatric health disparities, dissemination and implementation science and ethics related to digital mental health (DMH) technologies for underserved populations. The research plan seeks to integrate Teen Assess, Check, and Heal (TeACH) System into a pediatric primary care clinic treating teens and their families, as well as conduct a pilot trial to examine engagement, implementation and disparities outcomes. The application comes from an extremely strong candidate with 55 publications (30 first author) who is supported by an outstanding mentoring team and environment. The clear benefits of the research plan include the scalability of TeACH, use of a stakeholder informed approach to development, implementation in a pediatric primary care office, pilot randomized design for Aim 3, and the collection of quantitative and qualitative data; the preliminary data will likely support a future R01 application. The proposed career development plan is equally well designed, and it is well justified in the context of the candidate's previous training. Only minor weaknesses are noted, such as, the insufficient justification of the exclusion of teens with depression and the choice of the one-week follow-up period. Overall, this is an outstanding K08 application that will likely position the candidate to conduct high-impact, independent research to reduce mental health disparities in underserved populations.

DESCRIPTION (provided by applicant):
Mental health disorders are the most common disease of childhood. Yet, millions of teens do not receive mental health care. Most at risk are teens from underserved populations (e.g., low socioeconomic status; racial/ethnic and/or gender/sexual minority), who face a myriad of barriers to mental health screening and care. As such, traditional methods for reaching underserved teens with mental health disorders are not working, resulting in life-long health disparities and a significant public health impact. Consistent with the recommendations made in the NIMH National Advisory Mental Health Council Workgroup report, the goal of this K08 application is to use and adapt existing digital mental health technologies to advance the engagement, assessment, detection, treatment, and delivery of services for pediatric mental health. Specifically, the Accelerated Creation-to-Sustainment Model will guide the development and implementation of the Teen Assess, Check, and Heal (TeACH) System into a pediatric primary care clinic serving teens and families from the West Side of Chicago. In Aim 1, the PI and her mentorship team will collaborate with underserved teens (n=20) and their parents (n=20) to identify strategies to target top barriers to engagement as well as top ethical concerns and requirements for cultural relevance, usability, and usefulness of the TeACH System. In Aim 2, the plan for implementing the TeACH System will be refined through observations, interviews, and co-design workshops with pediatric primary care pediatricians and staff. In Aim 3, the TeACH System will be implemented into a primary care clinic and evaluated in a randomized trial for: 1) engagement and implementation outcomes; and 2) assessment of remediation of health disparities by analyzing differential outcomes (e.g., race, insurance status, individual perceptions of mental health) in a randomized trial. This innovative research will inform general digital mental health technology engagement adaptations needed for underserved teens and identify implementation practices to support the TeACH System in pediatric primary care settings. The PI and her mentorship team will also determine the feasibility and satisfaction of the TeACH System in preparation for the PI's planned expansion of the System across multiple primary care clinics in a future R01 proposal. The proposed research and career development plans logically build from the PI's foundational training in pediatrics/behavioral health, user-centered design, and mobile health (mHealth) evaluation to provide opportunities to gain knowledge and skills in: 1) pediatric health disparities; 2) dissemination and implementation science; and 3) ethics specific to deploying digital mental health technologies for underserved populations. Supported by an interdisciplinary team of experts and in institutional environment invested in supporting innovative initiatives to improve the mental and behavioral health of

underserved populations, this K08 will launch the PI into a successful career as an independent clinical scientist.

PUBLIC HEALTH RELEVANCE:
Millions of American teens do not receive appropriate mental health care; teens from underserved populations face the greatest barriers. The proposed research will harness existing digital mental health technologies, such as computerized assessments and mobile health [mHealth] apps, to develop a screening and treatment system which we will then implement and evaluate for engagement outcomes in a pediatric primary care clinic on the West Side of Chicago. The knowledge gained from this project will demonstrate the real-world engagement, acceptability, access, and use of these digital mental health technologies to decrease pediatric mental health disparities.

CRITIQUE 1

Candidate: 1
Career Development Plan/Career Goals /Plan to Provide Mentoring: 1
Research Plan: 2
Mentor(s), Co-Mentor(s), Consultant(s), Collaborator(s): 1
Environment and Institutional Commitment to the Candidate: 1

Overall Impact:
This is a resubmitted K08 application addressing an important topic (health disparities among low-income adolescents): standard computerized screening for adolescent mental health difficulties and randomizing to either DHT or psychoeducational handouts, conducted in an underserved area of Chicago. This is an extremely strong candidate with 55 publications (30 1st author) and a prior F, and a very strong (but large) mentoring team. An exciting element of this TEACH intervention is that it is highly scalable, requiring no effort or time from clinicians or office staff. Training goals include gaining knowledge and skills in: 1) pediatric health disparities; 2) dissemination and implementation science; and 3) ethics specific to deploying digital mental health technologies for underserved populations. Strengths of the research include addressing health disparities in underserved adolescents, use of a stakeholder informed approach to development, implementation in a pediatric primary care office, pilot randomized design for Aim 3, collection of quantitative and qualitative data. Concern includes need for greater justification that mHealth interventions can make a difference. There is also still a minor concern regarding sole focus on anxiety; however, there is a need to ensure that the proposed research is feasible and scaled to this funding mechanism. Otherwise, the resubmission is highly responsive to reviews, and this is a solid compelling application by a highly qualified candidate. The remaining concerns are addressable.

1. Candidate:
Strengths
- Very strong candidate with 55 publications (30 1st author), an early career award, and she had an F31. This is an exceptional level of productivity!
- Foundational training in the design and evaluation of digital mental health technologies (DMH), user-centered design principles and DMH impact on mental health symptoms in research contexts. DMH has the potential to be an effective approach to timely and accurate mental health screening and referral.

Weaknesses
- [None noted]

2. Career Development Plan/Career Goals & Objectives 1
Strengths

- Excellent career development plan.
- Each research aim has corresponding training goals.
- Mentors well detailed the specific activities listed each year.

Weaknesses
- [None noted]

3. Research Plan:
Strengths
- Addresses an important and significant topic—connecting underserved adolescents with mental health problems with evidence-based DHT.
- Scalable, given low burden on clinicians and staff.
- Partnership with primary care, which is a less stigmatizing setting vs mental health settings.
- Use of diverse teen and parent stakeholders to iteratively design and refine intervention, address barriers (e.g., stigma, mistrust, access).
- Digital mental health addresses barriers to face-to-face care.
- Implementation outcomes will be measured: staff and pediatricians from pediatric primary care will complete interviews and organizational assessments (e.g., observational studies of workflows, co-design workshops) will be conducted around routine well child visits.
- It is good that the application expanded recruitment to include 3 options, with text and email being alternatives to MyChart, in the likely event that a teen does not use MyChart or forgot pw.

Weaknesses
- The focus on anxiety only can be considered a limitation but was justified in design considerations. Unclear how comorbid depression or other commonly comorbid disorders will be considered.
- It is not fully clear if the mHealth intervention is truly powerful enough to have an effect. This discussion could have been stronger in the application. Still, this “light touch” intervention will be an improvement for teens who might have undiagnosed or otherwise untreated mental health difficulties.
- If adolescents with a disorder are referred for treatment, it is not clearly discussed how that might interfere with randomization.

4. Mentor(s), Co-Mentor(s), Consultant(s), Collaborator(s):
Strengths
- Mentorship team is very strong covering expertise in health disparities, implementation science, ethics of working with underserved participants.
- Though there is a large number of mentors, they each have specific roles and primary mentor will manage them.
- Primary mentor has established collaboration with all of the co-mentors and consultants.
- Most of mentors are located in Chicago which makes collaboration and relationship building easier.

Weaknesses
- It will be an important professional development experience for the candidate to learn through this process to manage multiple mentors, with help from the primary mentor.

5. Environment and Institutional Commitment to the Candidate:
Strengths
- Environment is very strong.
- Many relevant classes offered and included in the training plan.
- Centers with direct relevance to training plan.
- Letters of support from clinics, with whom team has longstanding relationships.

Weaknesses

- [None noted]

Study Timeline:
Strengths
- Reasonable.

Weaknesses
- [None noted]

Protections for Human Subjects:
Acceptable Risks and Adequate Protections
- Good plan for suicidal thoughts and behaviors.

Data and Safety Monitoring Plan (Applicable for Clinical Trials Only):
Acceptable.
- DSMB for Aim 3 RCT.

Inclusion Plans:
- Sex/Gender: Distribution justified scientifically
- Race/Ethnicity: Distribution justified scientifically
- For NIH-Defined Phase III trials, Plans for valid design and analysis:
- Inclusion/Exclusion Based on Age: Distribution justified scientifically

Vertebrate Animals:
Not Applicable (No Vertebrate Animals).

Resubmission:
- Highly responsive.

Training in the Responsible Conduct of Research
Acceptable.
Comments on Format (Required):
Comments on Subject Matter (Required):
Comments on Faculty Participation (Required; not applicable for mid- and senior-career awards):
Comments on Duration (Required):
Comments on Frequency (Required):

Resource Sharing Plans:
Not Applicable (No Relevant Resources).

Budget and Period of Support:
Recommend as Requested.

CRITIQUE 2

Candidate: 1
Career Development Plan/Career Goals /Plan to Provide Mentoring: 1
Research Plan: 3
Mentor(s), Co-Mentor(s), Consultant(s), Collaborator(s): 1
Environment and Institutional Commitment to the Candidate: 1

Overall Impact:

The proposed K08 application is designed to increase the candidate's knowledge and skills related to pediatric health disparities, dissemination and implementation science and ethics related to digital mental health (DMH) technologies for underserved populations. The proposed study on the use and adaptation of existing digital mental health technologies to reach underserved teen populations has tremendous potential for decreasing barriers to mental health care. The candidate has excellent prior training and experience and a track record of productivity in terms of peer-reviewed publications. The career development plan is solid and includes coursework on health care disparities, community health, health care ethics and cultural competence, and both quantitative and qualitative methods. The proposed training and preliminary data will inform a future R01 application that will use a system (TeACH system) for multiple mental health disorders within pediatric primary health care clinics. The primary mentor, co-mentors, consultants and collaborators have the expertise needed to mentor the candidate in the identified areas and to assist the candidate in the successful implementation of the three study aims. The three aims focusing on DMH engagement seem feasible given the timeframe and resources. Due to the prevalence of comorbidities, there is the need in real world settings to address multiple problems. While it makes sense methodologically in a small trial to start with one major domain (anxiety) when providing recommendations for mHealth apps to establish proof of concept, it is not fully clear if findings will be translatable to other domains/diagnoses for a future R01 addressing various problems. Specifically, it is not clearly justified why depression is excluded for Aim 3. However, data from K-CAT will help inform broader needs to be addressed in future trials and the data regarding engagement with the TeACH system will inform future research. The timeframe for one-week follow-up might have been more adequately clarified. Overall, the proposed career development plan for the candidate, as well as the proposed research plan, has tremendous potential for establishing the candidate as a leader in the field of DMH technologies for underserved youth.

1. Candidate:
Strengths
- Dr. Stiles-Shields has a solid foundation for the proposed application including serving as first author on 30 of the 55 peer-reviewed publications.
- Dr. Stiles-Shields is currently engaged in projects related to digital mental health and is serving as PI for two of these projects.

Weaknesses
- None.

2. Career Development Plan/Career Goals & Objectives:
Strengths
- The training plan to address the three goals (i.e., pediatric health disparities, dissemination and implementation science and ethics related to DMH for underserved populations) is sound.

Weaknesses
- None.

3. Research Plan:
Strengths
- Incorporating DMH among underserved teens who visit pediatric primary care clinics is innovative.
- Teens and their caregivers will be involved in the co-design of the prompts to target engagement.
- The team has a track record of integrating another program (BEC) into pediatric primary care centers which provides support for the feasibility of this study.
- Use of advisory panels with teens and parents to inform iterative adaptations of TeACH system.
- The TeACH system utilizes the K-CATS, a brief, computerized assessment and addresses multiple diagnoses.
- Utilizing mixed methods to address Aim 2 is a strength.

- A one-month pilot implementation will occur prior to the RCT.

Weaknesses

- The rationale for only including mHealth apps for anxiety is clearly stated. However, it is not fully clear if the findings related to engagement will be applicable to other disorders, such that this study would inform a future R01 targeting numerous disorders or is it possible that engagement differs based on disorder.
- Aim 1 includes the inclusion of depression and anxiety as these diagnoses are prevalent among youth. Thus, it is not clearly justified why depression is excluded in Aim 3.
- It is not fully clear if the immediate and one-week follow up assessments provide enough time for teens to engage in actual use. Why was the one-week timeframe chosen?
- Minor comment: the age range is 13 to 17, but Section 2.3 states that the minimum age is 12 years. It also seems that the planned ethnic categories should include all teens who assented (N = 326; those who complete the initial questionnaire) and not just those who complete questionnaires at both time points.

4. Mentor(s), Co-Mentor(s), Consultant(s), Collaborator(s):

Strengths

- The five mentors have the expertise needed, such as, technology-supported evidence-based mental health interventions for underserved youth, ethics, statistics, qualitative research, pediatric health disparities, dissemination and implementation science, and experience mentoring early-stage investigators.
- The five mentors are from diverse disciplines (psychiatry, sociology, nursing, pediatrics, psychology, and biostatistics).
- Consultants and collaborators provide additional expertise and support.
- The timeline is clear, and most coursework occurs in Years 1 and 2.

Weaknesses

- There is now a plan to have Dr. Karnik coordinate the team of co-mentors and collaborators regarding the mentorship. However, since future trials will most likely involve multiple co-investigators, consultants, community partners and assistants is there a way to harness the experience of Dr. Karnik to take a collaborative role in coordinating and managing mentorship?

5. Environment and Institutional Commitment to the Candidate:

Strengths

- Rush University Medical Center has coordinated efforts to expand community-based initiatives, including support for communities on the west side of Chicago.
- There are several centers, including the Center for Underserved Populations and Innovative Interventions, MacLean Center for Clinical Medical Ethics, The Center for Health Statistics and Center for Community Health Equity, and the Center for Dissemination and Implementation Science that may serve as resources for Dr. Stiles-Shields.
- Dr. Karnik has offered to fund needs to support this application that are not covered by the K award.

Weaknesses

- None.

Study Timeline:

Strengths

- Activities and quarterly time periods for each aim are clearly noted.

Weaknesses

- None.

Protections for Human Subjects:

Acceptable Risks and Adequate Protections

- The K-Cat will be administered while in direct contact with a licensed clinician. Procedures for assessing suicidal ideation are in place.

Data and Safety Monitoring Plan (Applicable for Clinical Trials Only):

Acceptable.

- DSMB in place for study in Aim 3.

Inclusion Plans:

- Sex/Gender: Distribution justified scientifically
- Race/Ethnicity: Distribution justified scientifically
- For NIH-Defined Phase III trials, Plans for valid design and analysis: Scientifically acceptable
- Inclusion/Exclusion Based on Age: Distribution justified scientifically

Vertebrate Animals:
Not Applicable (No Vertebrate Animals).

Training in the Responsible Conduct of Research:
Acceptable.
Comments on Format (Required):

- Appropriate.

Comments on Subject Matter (Required):

- Appropriate.

Comments on Faculty Participation (Required; not applicable for mid- and senior-career awards):

- Good.

Comments on Duration (Required):

- Appropriate.

Comments on Frequency (Required):

- Appropriate.

Resource Sharing Plans:
Not Applicable (No Relevant Resources).

Budget and Period of Support:
Recommend as Requested.

CRITIQUE 3

Candidate: 1
Career Development Plan/Career Goals /Plan to Provide Mentoring: 1
Research Plan: 2
Mentor(s), Co-Mentor(s), Consultant(s), Collaborator(s): 1
Environment and Institutional Commitment to the Candidate: 1

Overall Impact:
This K08 application aims to train the candidate in: 1) pediatric health disparities; 2) dissemination and implementation science; and 3) ethics related to deploying digital mental health (DMH) tools for underserved populations. To support this training, the candidate proposes to develop and implement the Teen Assess, Check, and Heal (TeACH) System in a pediatric setting. The aims of the study are to: 1) engage teens and parents in refining TEACH for underserved populations; 2) engage primary care stakeholders to help develop implementation procedures; and 3) conduct a randomized feasibility trial of TeACH in an urban pediatric primary care clinic. This candidate is extremely strong, and the

proposed training plan is well designed and justified in the context of her previous training. The proposed research is innovative and holds the potential to reduce mental health disparities. There are a few minor weaknesses to the application, but they are addressable. This award will position the candidate to conduct high-impact, independent research focused on applying DMH to reducing mental health disparities in underserved populations.

1. Candidate:
Strengths
- The candidate has an impressively strong publication record (55 peer-reviewed, 30 first authored).
- She has an excellent training background in telehealth, user-centered design principles, and mobile health evaluation.
- Her letters of recommendation are very positive and specific about her strengths.

Weaknesses
- None noted.

2. Career Development Plan/Career Goals & Objectives:
Strengths
- The career development plan is a logical extension of the candidate’s existing expertise.
- The training plan is detailed and involves a combination of mentorship, coursework, and fellowship experiences that are tied to the proposed research.

Weaknesses
- None noted.

3. Research Plan:
Strengths
- The proposed screening and digital mental health referral tool is innovative and a great way to increase access to care, particularly when housed within a primary care setting and build upon the feedback of community stakeholders.
- The application presents substantial support for the feasibility of the proposed study.

Weaknesses
- While it is a strength that participants from other clinics will participate in Aim 2, very little information is provided about them, including how many of them will take part and what they will be asked to do. Will they only take part in interviews? Or will they also be observed and/or take part in the co-design workshops? This is a minor weakness.
- While the tiered lottery system is an improvement, it seems another way to manage budget would be to specify a target sample and stick to it. Given the low-income populations being targeted here, incentivizing time spent on assessment measures for all participants seems a more respectful choice. This is a minor weakness.

4. Mentor(s), Co-Mentor(s), Consultant(s), Collaborator(s):
Strengths
- This is a strong mentoring and consultation team with clearly defined roles that relate to the proposed training goals.

Weaknesses
- None noted.

5. Environment and Institutional Commitment to the Candidate:
Strengths
- The candidate already has a faculty position and commitment from her institution seems high.

- The scientific environment at Rush University Medical Center is strong and the candidate has established collaborations with the data collection sites.

Weaknesses

- None noted.

Study Timeline:

Strengths

- The timeline is feasible.

Weaknesses

- [None noted]

Protections for Human Subjects:

Acceptable Risks and Adequate Protections

- Protections are appropriate.

Data and Safety Monitoring Plan (Applicable for Clinical Trials Only):

Acceptable.

- There are identified members for a DSMB.

Inclusion Plans:

- Sex/Gender: Distribution justified scientifically
- Race/Ethnicity: Distribution justified scientifically
- For NIH-Defined Phase III trials, Plans for valid design and analysis: Not applicable
- Inclusion/Exclusion Based on Age: Distribution justified scientifically
- All inclusions are justified.

Vertebrate Animals:

Not Applicable (No Vertebrate Animals).

Resubmission:

- The application is highly responsive to previous reviews.

Training in the Responsible Conduct of Research:

Acceptable.

Comments on Format (Required):

- A combination of in person and online.

Comments on Subject Matter (Required):

- Sufficiently broad.

Comments on Faculty Participation (Required; not applicable for mid- and senior-career awards):

- Faculty will be involved.

Comments on Duration (Required):

- More than 8 hours.

Comments on Frequency (Required):

- Over the life of the award.

Resource Sharing Plans:

Not Applicable (No Relevant Resources).

Budget and Period of Support:

Recommend as Requested.

THE FOLLOWING SECTIONS WERE PREPARED BY THE SCIENTIFIC REVIEW OFFICER TO SUMMARIZE THE OUTCOME OF DISCUSSIONS OF THE REVIEW COMMITTEE, OR REVIEWERS' WRITTEN CRITIQUES, ON THE FOLLOWING ISSUES:

PROTECTION OF HUMAN SUBJECTS: ACCEPTABLE. The K-Cat will be administered while in direct contact with a licensed clinician. Procedures for assessing suicidal ideation are in place. There is a DSMB for the study in Aim 3.

INCLUSION OF WOMEN PLAN: ACCEPTABLE

INCLUSION OF MINORITIES PLAN: ACCEPTABLE

INCLUSION ACROSS THE LIFESPAN PLAN: ACCEPTABLE. Adolescents are the focus of the intervention to be studied. Data are also collected from their parents and healthcare providers.

COMMITTEE BUDGET RECOMMENDATIONS: The budget was recommended as requested.

Footnotes for 1 K08 MH125069-01A1; PI Name: Stiles-Shields, Colleen

NIH has modified its policy regarding the receipt of resubmissions (amended applications).See Guide Notice NOT-OD-18-197 at https://grants.nih.gov/grants/guide/notice-files/NOT-OD-18-197.html. The impact/priority score is calculated after discussion of an application by averaging the overall scores (1-9) given by all voting reviewers on the committee and multiplying by 10. The criterion scores are submitted prior to the meeting by the individual reviewers assigned to an application, and are not discussed specifically at the review meeting or calculated into the overall impact score. Some applications also receive a percentile ranking. For details on the review process, see http://grants.nih.gov/grants/peer_review_process.htm#scoring.
